# Transcriptomics and physiology reveal the mechanism of potassium indole-3-butyrate (IBAK) mediating rice resistance to salt stress

**DOI:** 10.1186/s12870-023-04531-1

**Published:** 2023-11-16

**Authors:** Hang Zhou, Meiling Liu, Fengyan Meng, Dianfeng Zheng, Naijie Feng

**Affiliations:** College of Coastal Agricultural Sciences, Guangdong Ocean University, Zhanjiang, 524088 China

**Keywords:** Rice, Salt stress, Transcriptome, Physiology, Potassium indole-3-butyrate

## Abstract

**Background:**

IBAK, as a plant growth regulator, has broad application prospects in improving crop resistance to abiotic stress.

**Results:**

In this study, the regulation mechanism of IBAK on rice was revealed by physiology and transcriptomics by spraying 80 mg·L^−1^ IBAK solution on rice leaves at the early jointing stage under salt stress. The results showed that spraying IBAK solution on leaves under salt stress could significantly increase K^+^ content, decrease Na^+^ content, increase net photosynthetic rate (Pn), and increase the activity of catalase (CAT) and the contents of glutathione (GSH) and soluble protein in rice leaves. Using IBAK under salt stress increased the expression of plant hormone signal transduction pathway-related genes LOC4332548 and LOC4330957, which may help rice to more effectively sense and respond to plant hormone signals and enhance resistance to salt stress. In addition, the photosynthesis pathway-related genes LOC4339270, LOC4327150, and LOC4346326 were upregulated after using IBAK under salt stress, and the upregulation of these genes may be beneficial to improve the efficiency of photosynthesis and increase the photosynthetic capacity of rice. Regarding starch and sucrose metabolism pathway, spraying IBAK on leaves could promote the expression of sucrose synthesis-related gene LOC4347800 and increase the expression of starch synthesis-related genes LOC4330709 and LOC4343010 under salt stress. Finally, IBAK spraying resulted in the upregulation of multiple 50 S and 30 S ribosomal protein genes in the ribosome pathway, which may increase protein synthesis, help maintain cell function, and promote rice growth and development.

**Conclusion:**

The results of this study revealed the mechanism of IBAK mediating resistance to salt stress in rice.

**Supplementary Information:**

The online version contains supplementary material available at 10.1186/s12870-023-04531-1.

## Background

Rice is a short-day crop, suitable for growing in high-temperature and high-humidity environments [1]. It is one of the essential food crops for human beings and has a long history of cultivation and consumption. Salt stress is one of the major limiting factors in rice cultivation. Salt stress can lead to stunted growth of rice [2], decreased ability of roots to absorb water, imbalance of ions, and oxidative stress. As the global population continues to grow, it is expected to increase to 11.2 billion by 2100 [3], leading to increased demand for arable land and food production. According to report, the total area of salt-affected soils worldwide equals 17 million km^2^ [3], and these salt lands are important potential arable land resources. Through rational development and utilization, it is possible to break through the “ceiling” of cultivated area and achieve the expansion of cultivated land resources.

Under salt stress, the normal metabolism of plants is destroyed. A large amount of sodium ions and chloride ions enter the plant body, causing plant enzyme activity and protein function to be inhibited, which is not conducive to the normal life activities of plants. The inhibition of photosynthesis is one of the characteristics of plants in response to salt stress [4]. Under salt stress, plants cannot absorb enough water and mineral nutrients, and the decrease in stomatal conductance leads to a decrease in carbon dioxide absorption, which is not conducive to plant photosynthesis. Salt stress also leads to oxidative stress in plants [5], where large amounts of superoxide anions and hydrogen peroxide are produced, which damage cell membranes and affect the function of biomolecules such as DNA. Therefore, salt stress inhibits plants in many ways, and the superposition of these adverse effects ultimately limits crop growth and yield.

Plant growth regulators are a class of chemicals that can affect plant growth, development, and physiological and metabolic processes. The application of plant growth regulators in regulating plant growth and enhancing plant resistance to abiotic stress has been widely reported [6–9]. Meng et al. found that spraying exogenous hemin on leaves can alleviate NaCl stress by promoting photosynthesis and carbon metabolism in rice seedlings [10]. Huang et al. found that foliar application of prohexadione calcium can reduce salt stress damage to rice and increase total yield under salt stress conditions [11]. Zhang et al. reported that prohexadione calcium can improve the salt tolerance of rice seedlings by enhancing lodging resistance, reducing membrane damage, and enhancing the photosynthesis and antioxidant capacities of rice seedlings [12].

Indole-3-butyric acid (IBA) is mainly used to induce the formation of adventitious roots in plants [13]. In *Arabidopsis thaliana*, IBA induced AR-formation by conversion into IAA involving NO activity, and by a positive action on IAA-transport and ASA1/ASB1-mediated IAA-biosynthesis [14]. Wang et al. studied the mechanism of indole-3-butyric acid-induced adventitious root formation in softwood cuttings of the *Catalpa bungei* variety, and found that during adventitious root formation, the number of differentially expressed genes (DEGs) involved in glycolysis decreased, while the number of DEGs involved in phenylpropanoid biosynthesis increased [15]. Additionally, according to the report, spraying IBA can alleviate salt stress and improve photosynthetic pigment content in wheat [16]. At low (5.41 dSm^−1^) and moderate (8.44 dSm^−1^) salinity, IBA at 10 ppm enhanced the wheat seedling dry matter yield [17]. IBAK is the potassium salt form of IBA, and their growth-regulating effects are similar in many respects. The difference is that applying IBAK to crops would introduce potassium ions, which have been widely reported to improve plant salt stress resistance [18–20]. A previous study found that exogenous IBAK can reduce drought stress damage to soybean roots at the seedling stage [21]. Therefore, there is a specific scientific basis for studying the role of IBAK in alleviating rice salt stress.

Up to now, very few studies have been on applying IBAK on major crops, especially in rice under salt stress. The regulation mechanism of IBAK on rice under salt stress is still unclear. Therefore, revealing the mechanism of IBAK to alleviate salt stress damage in rice from the perspective of physiology and transcriptomics is significant in filling the research gap in this field.

## Results

### The changes in ion content in rice leaves

As shown in Fig. S1, the contents of K^+^, Na^+^, and Cl^−^ in rice leaves were significantly increased under salt stress. Compared with the control (CKY), the IBAKY showed a significant increase in K^+^ content, a significant decrease in Na^+^ content, and no significant change in Cl^−^ content.

### The changes in photosynthesis-related indicators in rice leaves

As shown in Fig. S2 , the Pn, transpiration rate (Tr), and stomatal conductance (Gs) were significantly reduced in CKY compared to CK, indicating that salt stress significantly inhibited rice photosynthesis. After spraying IBAK under salt stress, the Pn, Tr, and Gs in IBAKY were improved compared with CKY, and the Pn in IBAKY was significantly higher than that in CKY.

### The changes in antioxidant enzyme activity in rice leaves

This study found that salt stress significantly increased the activity of superoxide dismutase (SOD) and peroxidase (POD) in rice leaves. Compared with the control (CKY), the IBAKY showed an increasing trend in POD and ascorbate peroxidase (APX) activity, and the CAT activity was significantly higher than that of CKY (Fig. S3).

### The changes in GSH and soluble protein contents in rice leaves

In this study, salt stress significantly increased the GSH content in rice leaves. The IBAKY showed the significantly higher levels of GSH and soluble protein than the control (CKY) (Fig. S4).

### Transcriptome sequencing and analysis

#### Data quality summary

A summary of data quality was shown in Table S1. After filtering out raw data, checking the sequencing error rate and distribution of GC content, and the clean reads were obtained for subsequent analysis. The average clean bases of the 9 samples in this study were 6.51G; Q20 was greater than 97.21%; Q30 was greater than 92.72%. The statistical results of reads mapping to the reference genome showed that the total map was greater than 90.62%, and the unique map was greater than 88.57% (Table S2). In addition, this study randomly selected several DEGs for qRT-PCR testing, and the results confirmed the reliability of RNA-seq data (Table S3).

#### DEGs statistics

**Fig. 1:**
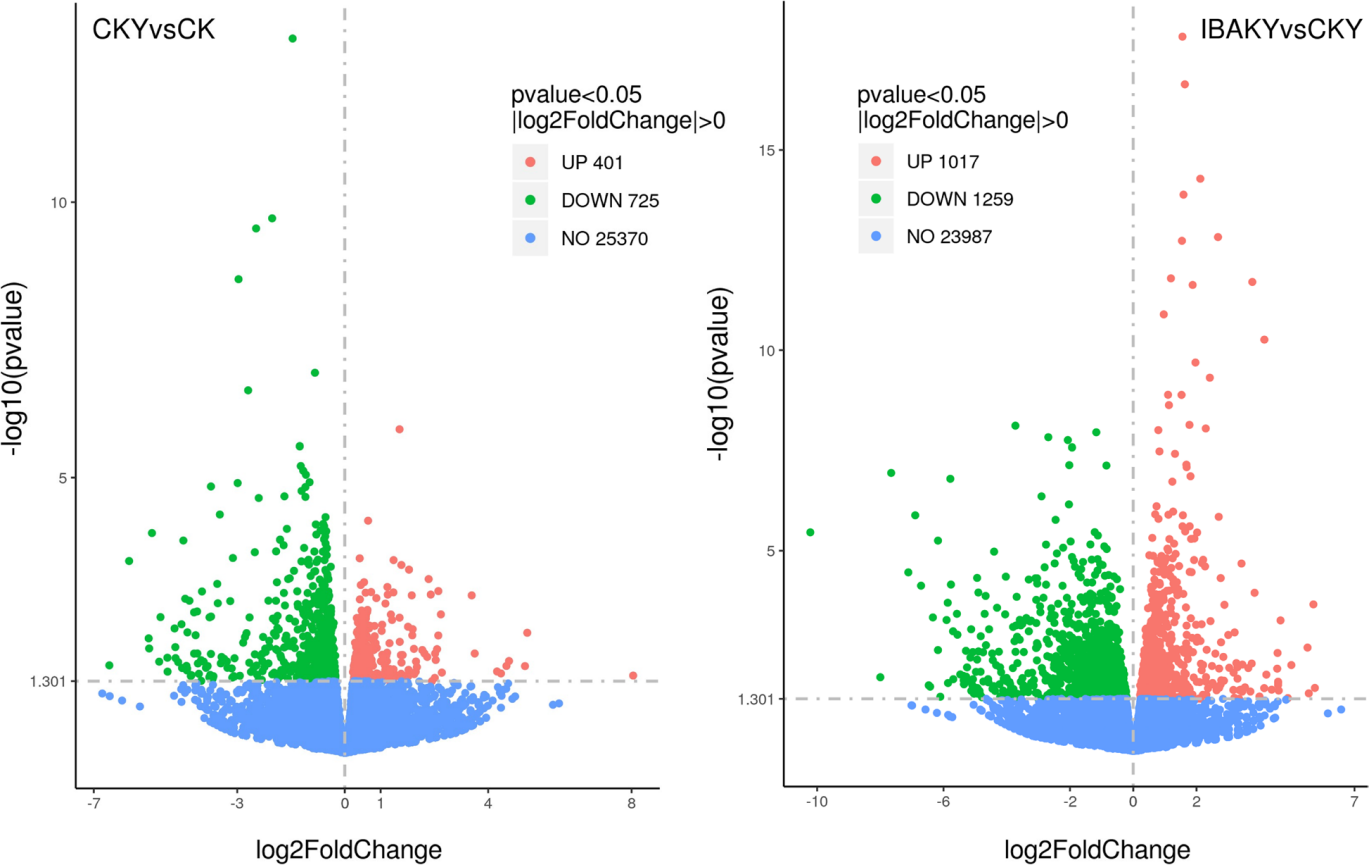
Volcano plot of DEGs. The horizontal axis represents the log2FoldChange value, and the vertical axis represents the -log10pvalue. CK, under freshwater conditions; CKY, under salt stress conditions; IBAKY, IBAK treatment under salt stress conditions

The number of DEGs (including upregulated and downregulated) in each comparison group and the criteria for screening differences were shown in Table S4. A total of 1126 DEGs were identified in the CKY vs. CK comparison group, including 401 upregulated and 725 downregulated genes. In the IBAKY vs. CKY comparison group, a total of 2276 DEGs were identified, including 1017 upregulated and 1259 downregulated genes (Fig. 1; Tables S4, S5 and S6).

#### GO enrichment analysis

**Fig. 2:**
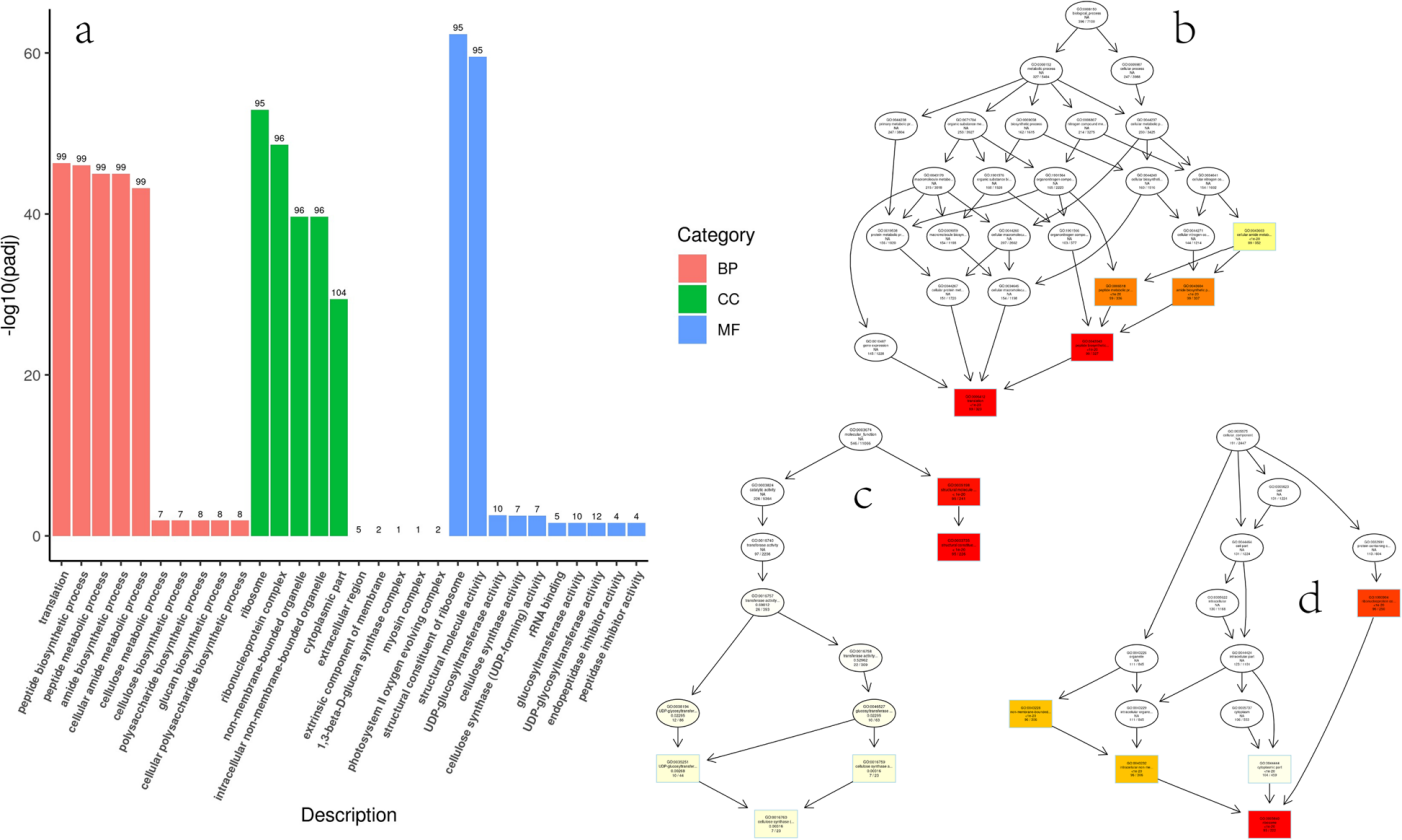
GO enrichment analysis histogram and DAG diagram in CKY vs. CK. **a** GO enrichment analysis histogram; the abscissa is GO term, and the ordinate is the significance level of GO term enrichment, represented by -log10(padj). **b**, **c**, and **d** GO enrichment analysis DAG diagram; **b**, BP; **c**, MF; **d**, CC; each node represents a GO term; the box represents the GO with an enrichment degree of TOP5; the depth of the color represents the enrichment degree, and the redder the color, the higher the enrichment degree; the term name and the padj of the enrichment analysis are displayed on each node. BP, biological process; CC, cellular component; MF, molecular function

GO function enrichment took padj less than 0.05 as the threshold of significant enrichment. The results of GO enrichment analysis showed that the most abundant DEGs were classified into translation, peptide biosynthetic process, peptide metabolic process, amide biosynthetic process, and cellular amide metabolic process in BP in CKY vs. CK; among them, translation was the most significantly enriched term. In CC, most DEGs were classified into ribosome, ribonucleoprotein complex, non-membrane-bounded organelle, intracellular non-membrane-bounded organelle, and cytoplasmic part; ribosome was the most significantly enriched term. In MF, DEGs were mainly classified into structural constituent of ribosome and structural molecule activity, and the significance of structural constituent of ribosome was the highest (Fig. 2; Table S7).

**Fig. 3:**
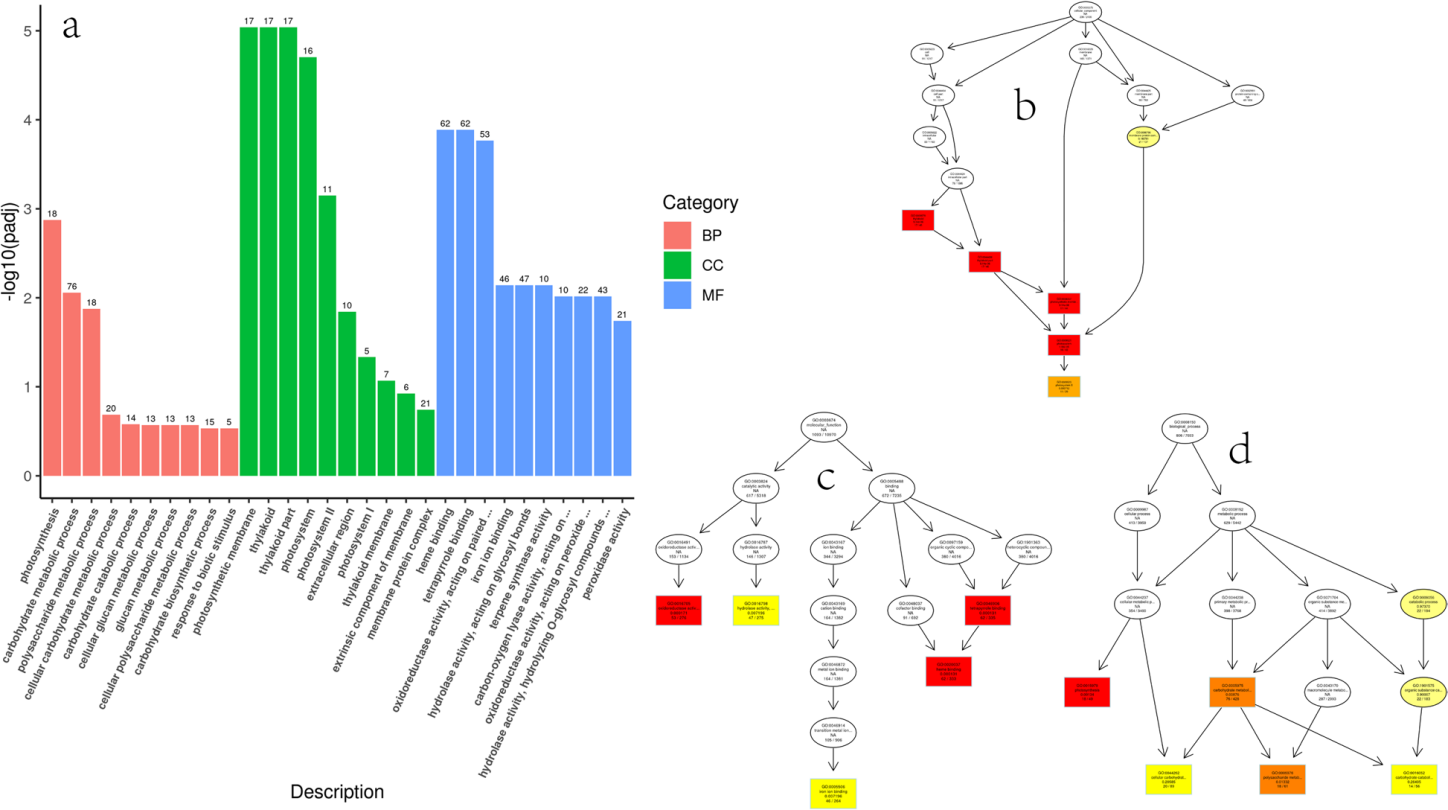
GO enrichment analysis histogram and DAG diagram in IBAKY vs. CKY. **a** GO enrichment analysis histogram; the abscissa is GO term, and the ordinate is the significance level of GO term enrichment, represented by -log10(padj). **b**, **c**, and **d** GO enrichment analysis DAG diagram; **b**, CC; **c**, MF; **d**, BP; each node represents a GO term; the box represents the GO with an enrichment degree of TOP5; the depth of the color represents the enrichment degree, and the redder the color, the higher the enrichment degree; the term name and the padj of the enrichment analysis are displayed on each node. BP, biological process; CC, cellular component; MF, molecular function

In IBAKY vs. CKY, most DEGs were classified into carbohydrate metabolic process in BP; the most significantly enriched term was photosynthesis. In CC, photosynthetic membrane, thylakoid, and thylakoid part were the most significantly enriched terms. Heme binding, tetrapyrrole binding, and “oxidoreductase activity, acting on paired donors, with incorporation or reduction of molecular oxygen” were the most significant terms containing the most DEGs in MF (Fig. 3; Table S8).

#### KEGG enrichment analysis

**Fig. 4:**
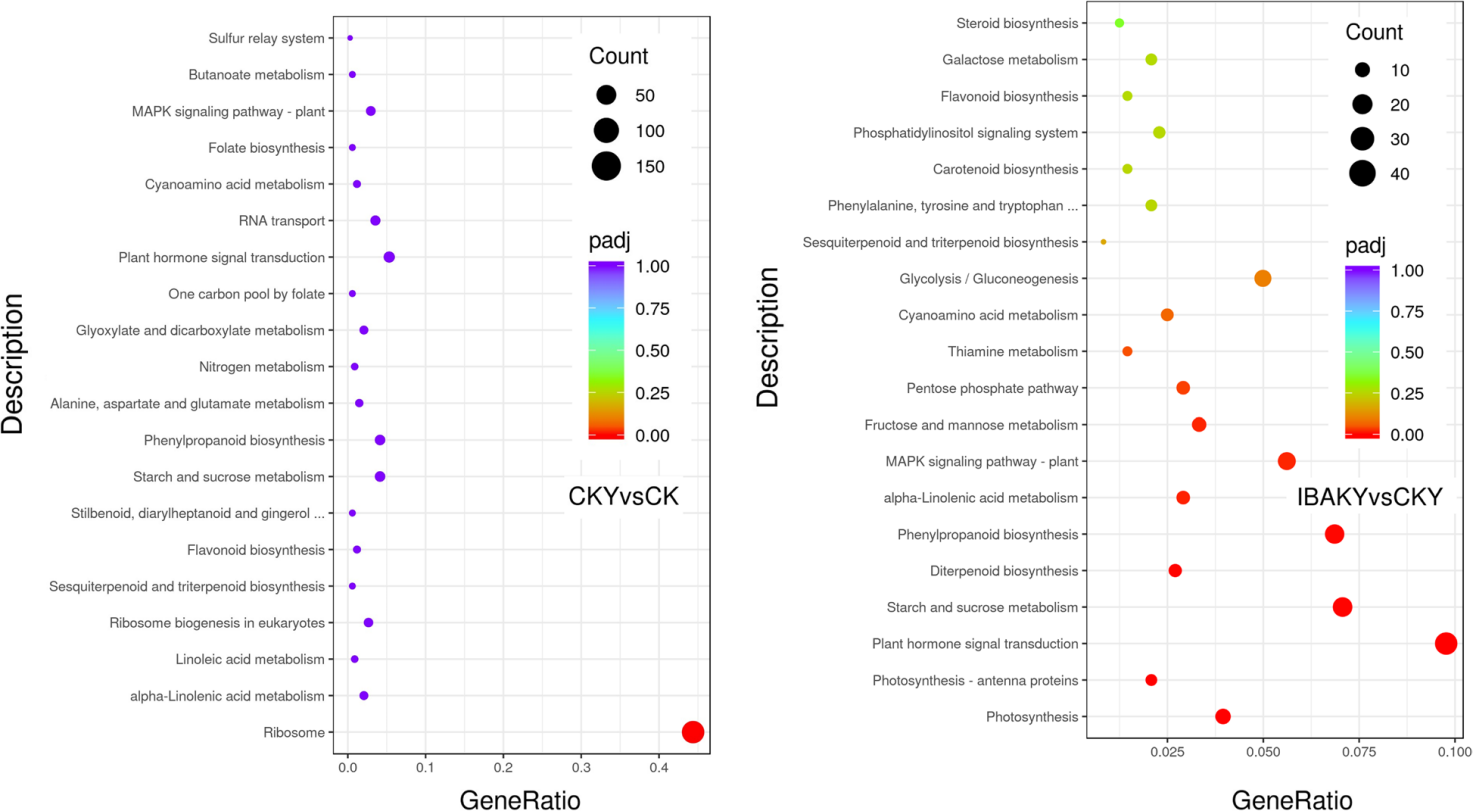
Scatter plot of KEGG enrichment. The horizontal axis represents the ratio of the number of differentially expressed genes annotated to the KEGG pathway to the total number of differentially expressed genes, and the vertical axis represents the KEGG pathway. The size of the points represents the number of genes annotated to the KEGG pathway. The color ranges from red to purple, representing the significance of the enrichment

KEGG pathway enrichment took padj less than 0.05 as the threshold of significant enrichment. In this study, the most significant 20 KEGG pathways were selected. Ribosome was the only pathway significantly enriched in the CKY vs. CK comparison group. Specifically, this study found that a total of 150 DEGs were enriched in the ribosome pathway, of which 1 DEG was upregulated, and 149 DEGs were downregulated. In addition, in the IBAKY vs. CKY comparison group, 11 pathways were significantly enriched in this study, and the most significant pathways were photosynthesis, photosynthesis - antenna proteins, and plant hormone signal transduction (Fig. 4; Tables S9 and S10).

#### DEGs involved in the photosynthesis, photosynthesis - antenna proteins, plant hormone signal transduction, starch and sucrose metabolism, and pentose phosphate pathway

This study focused on the discussion and analysis of photosynthesis, photosynthesis - antenna proteins, plant hormone signal transduction, starch and sucrose metabolism, and pentose phosphate pathway because these pathways play an essential role in the process of plant response to salt stress.

**Fig. 5:**
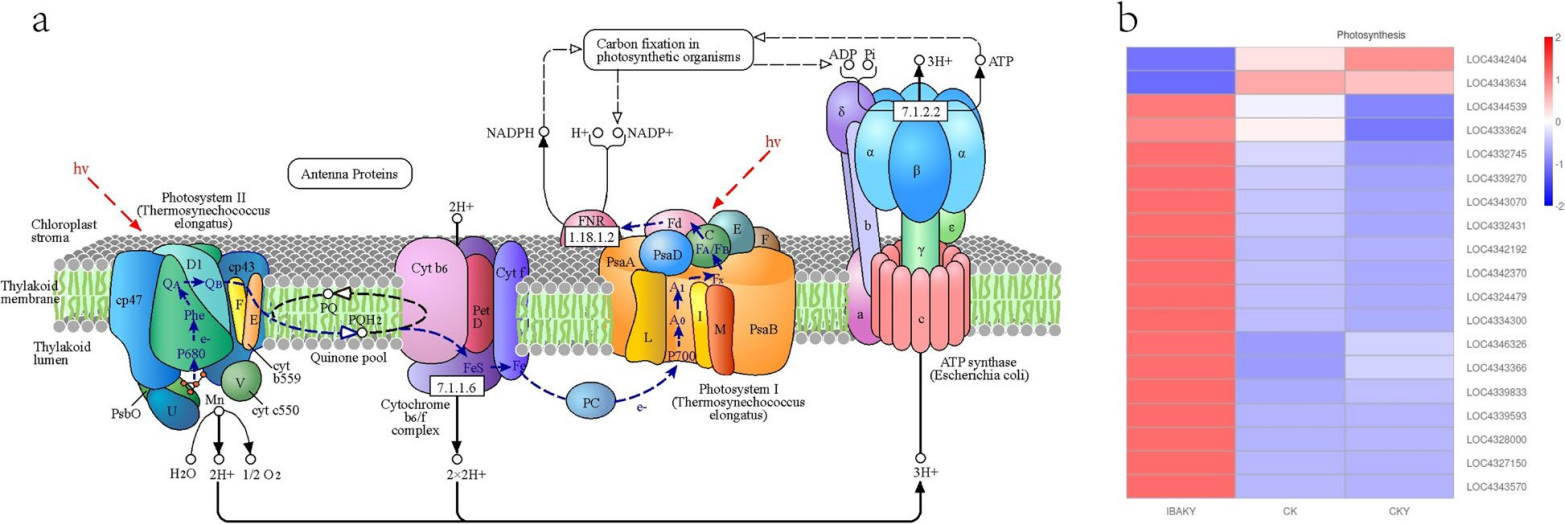
Heat map of gene expression in the photosynthesis pathway. **a** copyright permission of KEGG has been obtained

In this study, a total of 19 DEGs were found to be enriched in the photosynthesis pathway in the IBAKY vs. CKY comparison group, of which 17 were upregulated, and 2 were downregulated. Some key genes such as LOC4342370 (oxygen-evolving enhancer protein 2, chloroplastic), LOC4324479 (oxygen-evolving enhancer protein 1, chloroplastic), LOC4339270 (photosystem II reaction center W protein, chloroplastic-like), LOC4327150 (photosystem II reaction center W protein, chloroplastic-like), LOC4332745 (photosystem II repair protein PSB27-H1, chloroplastic), LOC4346326 (photosystem I reaction center subunit II, chloroplastic), LOC4343070 (photosystem I reaction center subunit IV, chloroplastic), LOC4334300 (photosystem I reaction center subunit III, chloroplastic), LOC4343366 (ATP synthase subunit gamma, chloroplastic), and LOC4332431 (ATP synthase subunit b’, chloroplastic) were upregulated (Fig. 5).

**Fig. 6:**
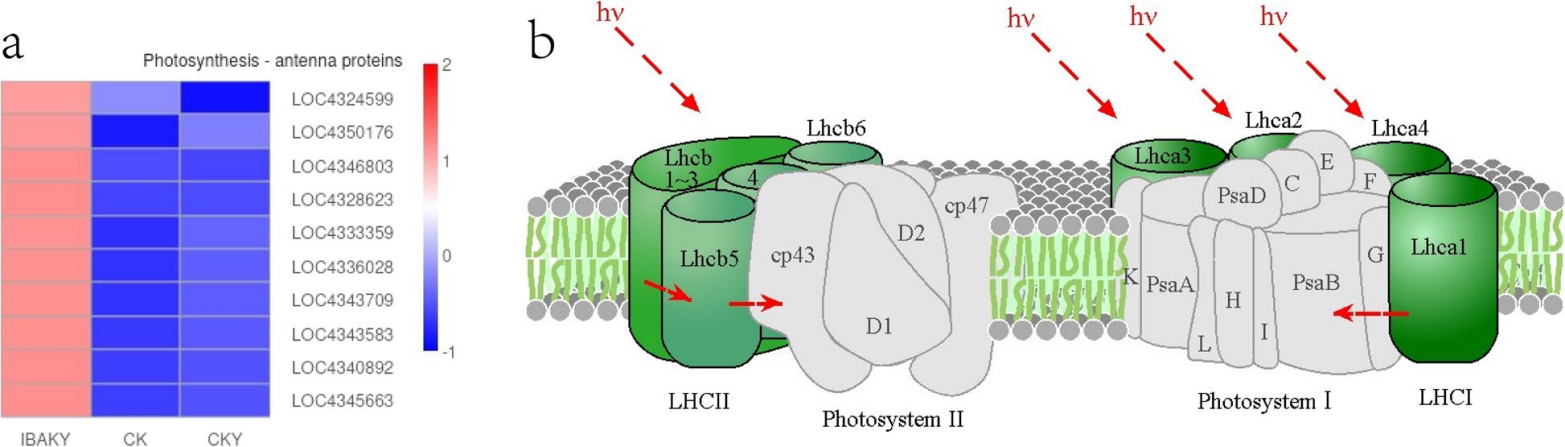
Heat map of gene expression in the photosynthesis - antenna proteins pathway. **b** copyright permission of KEGG has been obtained

This study found a total of 10 DEGs were enriched in the photosynthesis - antenna proteins pathway in IBAKY vs. CKY, and all these 10 genes were upregulated. Almost all of these genes were chlorophyll a-b binding protein genes, such as LOC4324599 (chlorophyll a-b binding protein 2, chloroplastic-like), LOC4346803 (chlorophyll a-b binding protein 1, chloroplastic-like), LOC4328623 (chlorophyll a-b binding protein 8, chloroplastic), LOC4336028 (chlorophyll a-b binding protein CP24, chloroplastic), LOC4343583 (chlorophyll a-b binding protein CP29.1, chloroplastic), LOC4340892 (chlorophyll a-b binding protein 1B-21, chloroplastic), LOC4345663 (chlorophyll a-b binding protein P4, chloroplastic), LOC4333359 (chlorophyll a-b binding protein, chloroplastic-like), and LOC4350176 (chlorophyll a-b binding protein CP26, chloroplastic) (Fig. 6).

**Fig. 7:**
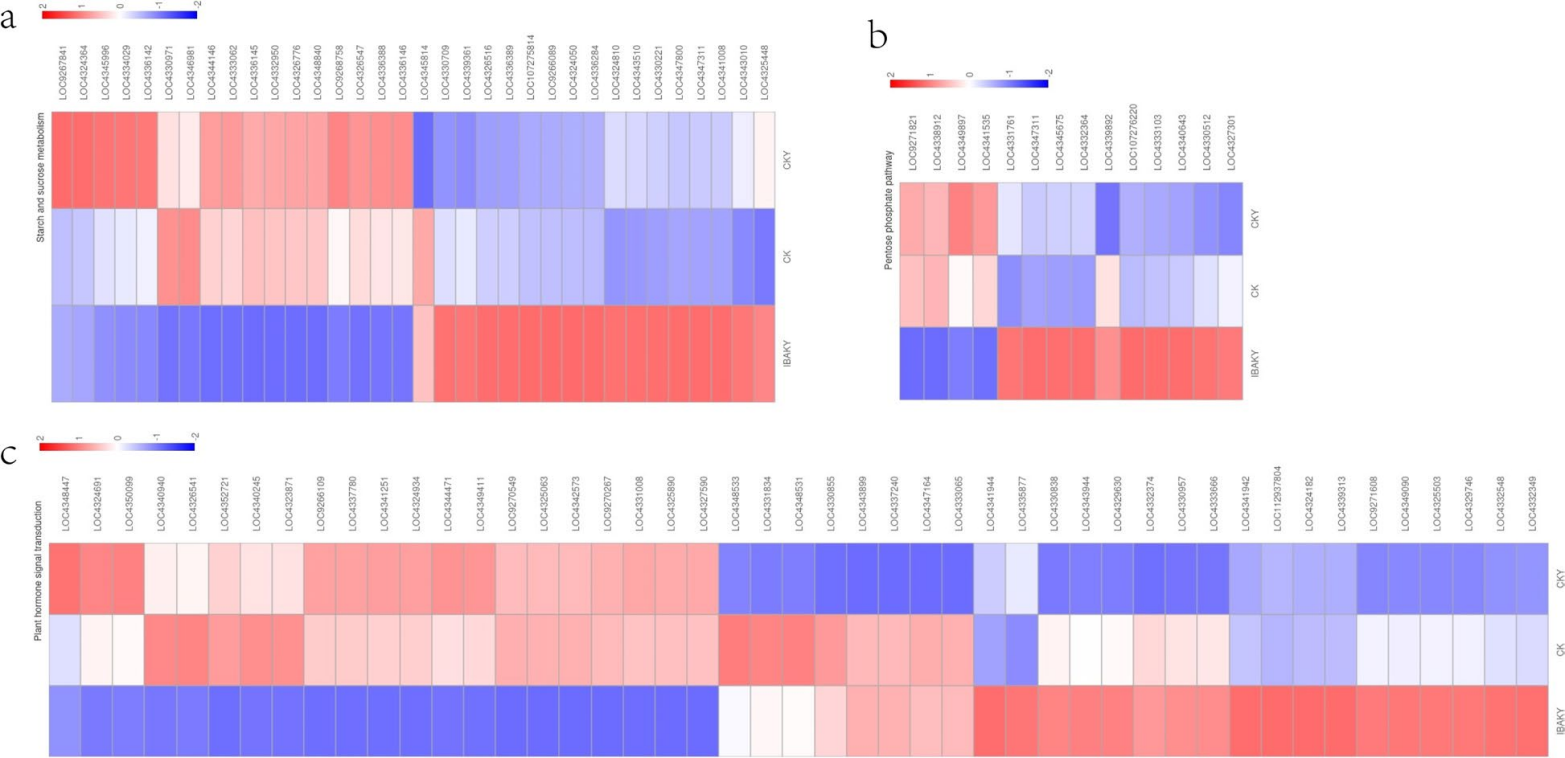
Heat map of gene expression in different pathways. **a** starch and sucrose metabolism pathway; **b** pentose phosphate pathway; **c** plant hormone signal transduction pathway

A total of 47 DEGs were enriched in the plant hormone signal transduction pathway in IBAKY vs. CKY, of which 26 were upregulated, and 21 were downregulated. Specifically, this study found that genes LOC4332548 (abscisic acid receptor PYL4), LOC4330957 (mitogen-activated protein kinase kinase 5), and LOC4343899 (protein TIFY 10b-like) were upregulated (Fig. 7c).

In the starch and sucrose metabolism pathway, 34 DEGs were found to be enriched in this study, of which 17 were upregulated, and 17 were downregulated. Some essential genes such as LOC4347800 (UTP–glucose-1-phosphate uridylyltransferase), LOC4330709 (soluble starch synthase 2–2, chloroplastic/amyloplastic-like), and LOC4343010 (granule-bound starch synthase 1b, chloroplastic/amyloplastic) were upregulated (Fig. 7a).

In addition, this study found that 14 DEGs were enriched in the pentose phosphate pathway in CKY and IBAKY, of which 10 were upregulated, and 4 were downregulated. Both LOC4331761 (ribulose-phosphate 3-epimerase, chloroplastic-like) and LOC4332364 (fructose-1,6-bisphosphatase, chloroplastic-like) were upregulated after using IBAK under salt stress (Fig. 7b).

#### Co-expressed gene analysis

Co-expression Wayne analysis can compare gene expression under different conditions. By studying co-expressed genes, we can better understand the roles and interrelationships of these genes in biological processes. The results of this study found 17,741 co-expressed genes among the three treatments.

We further performed KEGG enrichment analysis on these co-expressed genes. The results of KEGG enrichment analysis showed that ribosome, spliceosome, and RNA transport were the most significantly enriched pathways; co-expressed genes were mainly enriched in ribosome, carbon metabolism, and biosynthesis of amino acids pathway.

**Fig. 8:**
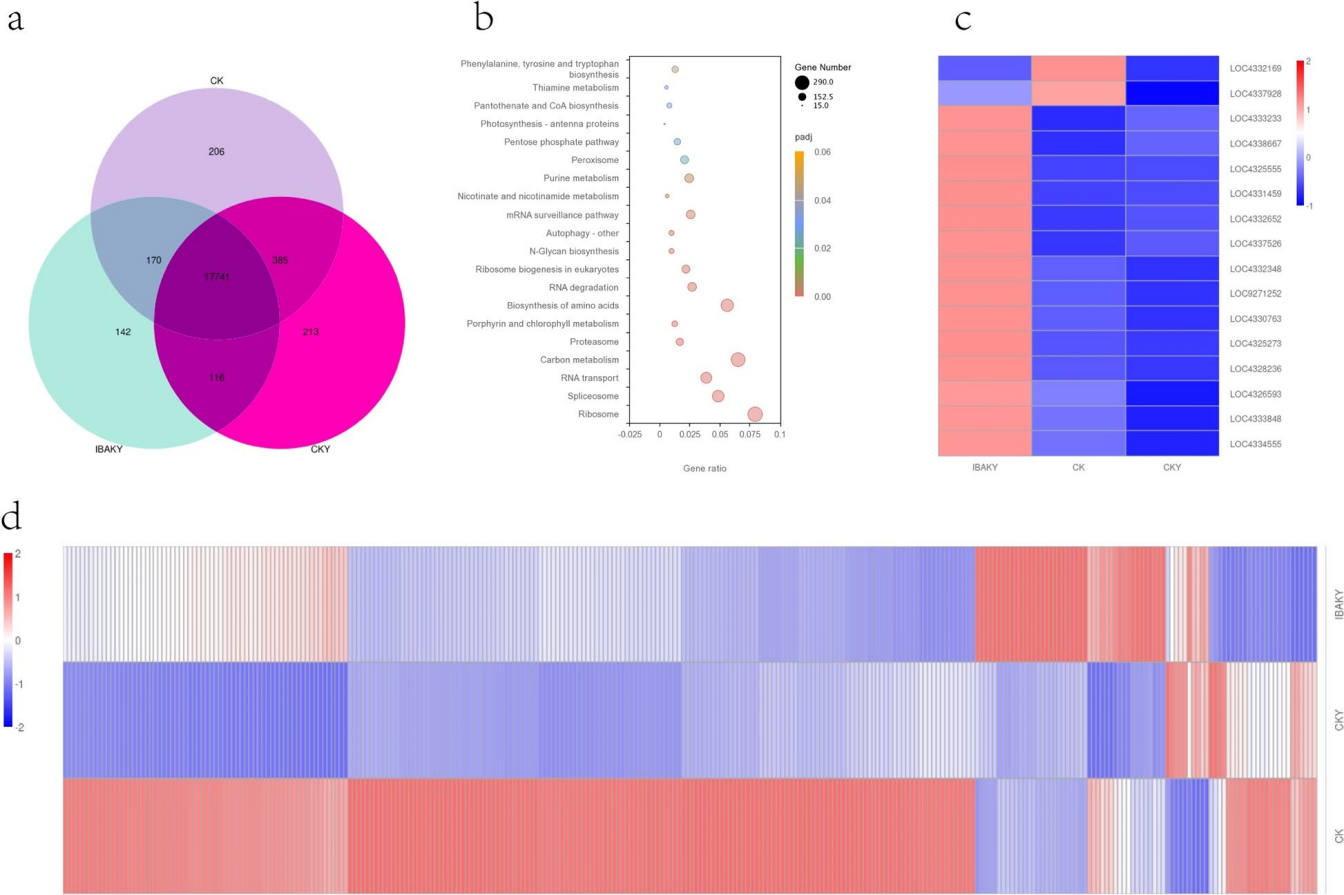
Co-expression gene analysis under different contexts. **a** Venn diagram of co-expressed genes; **b** KEGG enrichment analysis bubble plot of co-expressed genes; **c** heat map of highly expressed co-expressed genes in the ribosome pathway; **d** heat map of all co-expressed genes in the ribosome pathway

In this study, ribosome pathway was the most significantly enriched pathway. The higher the significance of the pathway, the higher the enrichment degree of the genes in this pathway; that is, the higher the degree of functional aggregation of these genes. This study found 290 co-expressed genes were detected to be enriched in the ribosome pathway, and among these 290 co-expressed genes, we screened a total of 16 highly expressed co-expressed genes (Fig. 8).

## Discussion

### Physiological response of rice to IBAK under salt stress

The results of this study showed that after spraying IBAK solution, K^+^ content in rice leaves significantly increased, and Na^+^ content significantly decreased under salt stress, which may help rice maintain ion balance under salt stress and alleviate the effects of salt stress damage on rice growth and development. Usually, salt stress causes plant roots to absorb excessive Na^+^, resulting in an imbalance of the K^+^/Na^+^ ratio and causing plants to suffer from stress damage. Increasing K^+^ content and reducing Na^+^ content in plants help plants adapt to the environment and maintain normal growth and development under salt stress conditions. Meawhile, this study measured rice photosynthesis-related indicators and found that salt stress significantly reduced Pn, Tr, and Gs values, resulting in an inhibitory effect on rice photosynthesis. However, interestingly, foliar spraying IBAK can effectively improve rice leaves Pn under salt stress. Photosynthesis is the basis of plant survival and reproduction, and is one of the main ways for plants to obtain energy. Foliar spraying IBAK promoted the photosynthesis of rice under salt stress, which was of great significance to increase the accumulation of photosynthetic products and increase rice yield under salt stress.

Furthermore, this study measured the activity of antioxidant enzymes in rice leaves. Under salt stress, the POD and APX activities in rice leaves tended to increase after spraying the IBAK solution. POD can convert hydrogen peroxide (H_2_O_2_) into water (H_2_O) and oxygen (O_2_) [22]. APX is a kind of peroxidase with AsA as the electron donor, and it is an important enzyme to remove H_2_O_2_ [23]. Therefore, the increased activity of POD and APX could help rice cope with oxidative stress under salt stress. In addition, this study found that the activity of CAT was significantly improved after using IBAK under salt stress. CAT is another antioxidant enzyme that breaks H_2_O_2_ into H_2_O and O_2_ [24]; improving its activity can further enhance the adaptability of rice to salt stress.

In addition to the abovementioned changes in antioxidant enzyme activity, this study found that applying IBAK under salt stress can significantly increase rice leaves’ GSH and soluble protein content. GSH is an important antioxidant substance that has the effect of removing oxidative stress substances. Applying IBAK under salt stress promoted the accumulation of GSH in rice leaves, which was beneficial to increase the antioxidant capacity of rice. At the same time, an increase in soluble protein content can help plants cope with stress environments, improving salt tolerance and adaptability.

### Regulation of plant hormone signal transduction pathway-related gene expression by IBAK in rice under salt stress

Plant hormone signal transduction plays a crucial role in plant defense mechanisms against various biotic and abiotic stresses. ABA is known as a stress hormone. It is involved in various physiological processes in plants, such as the responses to environmental stresses, making plants better adapt to the environment. Many studies showed that plants’ ABA levels would increase under salt stress [25, 26]. An increase in ABA triggers signaling cascades, and ABA receptors such as PYL4 play an essential role in mediating these responses. The gene LOC4332548 was described as abscisic acid receptor PYL4. In this study, the expression level of LOC4332548 in rice decreased under salt stress, while spraying IBAK on leaves under salt stress increased the expression level of LOC4332548 compared with CKY. Higher level of LOC4332548 expression may indicate a higher abundance of abscisic acid receptor PYL4, which may enhance the plant’s ability to sense and respond to ABA signals more effectively.

Transcriptome sequencing and q-PCR results showed that LOC4330957, described as mitogen-activated protein kinase kinase 5, was upregulated under salt stress after using IBAK. Overexpression of mitogen-activated protein kinase kinase 5 (MKK5) in wild-type *Arabidopsis* plants enhanced their tolerance to salt treatment, while *mkk5* mutant exhibited hypersensitivity to salt stress in germination on salt-containing media [27]. This suggested that the upregulation of LOC4330957 may be one of the reasons why IBAK improved salt tolerance in rice. In addition, the gene LOC4343899, described as protein TIFY 10b-like, was upregulated in this study. According to the report, *OsTIFY10b* was strongly induced by drought, salt, and cold in rice [28]. *OsTIFY10b*-transgenic plants exhibited higher total grain yield than wild-type plants, owing to fewer aborted caryopses [29].

### Regulation of photosynthesis pathway-related gene expression by IBAK in rice under salt stress

The study of IBAK in improving the photosynthetic ability of crop has been reported [30]. This study focused on the effect of IBAK on the expression of key photosynthesis-related genes in rice under salt stress. The results of KEGG enrichment analysis showed that the two pathways with the highest significance were in the IBAKY vs. CKY comparison group. This meant that spraying IBAK had an essential effect on the photosynthesis of rice leaves under salt stress. This study found that 19 DEGs were enriched in the photosynthesis pathway in the IBAKY vs. CKY comparison group, of which 17 DEGs were upregulated. The gene LOC4342370 is “oxygen-evolving enhancer protein 2, chloroplastic”; LOC4324479 is “oxygen-evolving enhancer protein 1, chloroplastic”. Both genes were upregulated after using IBAK under salt stress and were at high expression levels in CKY and IBAKY. 

In plants, photosystem II (PSII) uses the energy absorbed from light to split water and transfer the released electrons to plastoquinone. Photosystem II reaction center W protein has the function of stabilizing dimeric PSII, and no dimeric PSII supracomplex could be detected in the transgenic *Arabidopsis thaliana* plants lacking the PsbW protein [31]. In this study, the expression levels of genes LOC4339270 and LOC4327150 were detected to be upregulated after spraying IBAK under salt stress, and both genes were described as “photosystem II reaction center W protein, chloroplastic-like”. The upregulation of LOC4339270 and LOC4327150 expression levels in this study may lead to an increased abundance of photosystem II reaction center W protein, contributing to the stabilization of the dimeric PSII complex.

PSII is particularly vulnerable to damage due to exposure to high light intensity during photosynthesis. The repair process is essential to maintain the efficiency and function of the PSII under a variety of environmental conditions. Chen et al. found that recovery of PSII activity after photoinhibition was delayed in the *psb27* mutant in *Arabidopsis thaliana* under high-light-illumination, suggesting that Psb27 is required for efficient recovery of the photodamaged PSII complex [32]. Besides this high-light-illumination stress, the PSII complex may become damaged by other environmental stresses, such as salt stress. The gene LOC4332745 is “photosystem II repair protein PSB27-H1, chloroplastic”. This study found that the expression level of gene LOC4332745 was upregulated after using IBAK under salt stress. The upregulation of LOC4332745 in this study may be more conducive to the recovery of PSII activity in rice leaves under salt stress.

 In addition, gene LOC4346326 is “photosystem I reaction center subunit II, chloroplastic”. In the photosystem I (PSI) reaction center, photosystem I reaction center subunit II forms complexes with ferredoxin and ferredoxin-oxidoreductase. The gene LOC4343070 is “photosystem I reaction center subunit IV, chloroplastic”, which assists in the docking of the ferredoxin to PSI and interacts with ferredoxin-NADP oxidoreductase. The gene LOC4334300 is “photosystem I reaction center subunit III, chloroplastic”, participating in efficiency of electron transfer from plastocyanin to P700. These genes were all upregulated in this study under salt stress after applying IBAK. The upregulation of these genes may increase photosynthetic efficiency and productivity, which can benefit plants by providing more energy for plant growth, reproduction, and other metabolic processes.

In addition to the above, the upregulated LOC4343366 and LOC4332431 were described as “ATP synthase subunit gamma, chloroplastic” and “ATP synthase subunit b’, chloroplastic”, respectively. During photosynthesis, the ATP synthase subunit gamma and ATP synthase subunit b’ work with other subunits of the ATP synthase complex to generate ATP.

At the same time, this study found that 10 DEGs in the IBAKY vs. CKY comparison group were enriched to the photosynthesis - antenna proteins pathway, and these genes were all upregulated. These genes are almost all various chlorophyll a-b binding proteins. Chlorophyll a-b binding protein is an essential part of the light harvesting complex (LHC). LHC captures light energy and transfers it to the reaction centers of photosystems. Upregulation of these genes in this study can help rice maintain or increase photosynthetic efficiency under salt stress.

### Regulation of starch and sucrose metabolism pathway-related gene expression by IBAK in rice under salt stress

Under abiotic stress conditions, the normal metabolic processes of plants may be disrupted. Starch and sucrose metabolism can help plants cope with stress conditions by providing energy and carbon storage. Under salt stress, intracellular osmoregulation is imbalanced. In this process, sucrose plays a role in improving plants’ osmotic adjustment ability, enhancing plants’ tolerance to salt stress. Gene LOC4347800 was described as UTP–glucose-1-phosphate uridylyltransferase, which catalyzes the formation of UDP-glucose. UDP-glucose is a substrate for sucrose phosphate synthase to produce sucrose-6-phosphate, which is dephosphorylated to sucrose. This study found that the gene LOC4347800 was upregulated after using IBAK under salt stress, which may increase the synthesis of UDP-glucose, and this may further promote the synthesis of sucrose, which helps rice adapt to adversity and promotes growth and development.

Under environmental stress, the energy consumption of plants increases, so the synthesis and accumulation of starch becomes more critical. This study found that genes LOC4330709 (soluble starch synthase 2–2, chloroplastic/amyloplastic-like) and LOC4343010 (granule-bound starch synthase 1b, chloroplastic/amyloplastic-like) in IBAKY were significantly upregulated compared with CKY. Soluble starch synthase 2–2 may contribute to the deposition of transient starch in chloroplasts of leaves. Granule-bound starch synthase 1b is also one of the key enzymes in the starch synthesis process. The significant upregulation of these two genes in this study is of great significance and may play a positive role in the adaptation of rice to salt stress.

### Regulation of pentose phosphate pathway-related gene by IBAK in rice under salt stress

Pentose phosphate pathway plays a crucial role in the biosynthesis of nucleotides, amino acids and lipids, and in defense against oxidative stress. Salt stress causes oxidative damage to plants, producing many reactive oxygen free radicals, which damage cell membranes, proteins, and nucleic acids. NADPH is an important reduced coenzyme in cells, and more NADPH can increase the reducing ability of plants to help scavenge reactive oxygen free radicals. In addition, under salt stress, plants need more pentose sugars for the synthesis and repair of cellular nucleic acids and other biomolecules to maintain the normal function and growth of cells.

In this study, LOC4331761 and LOC4332364 had higher expression levels in CKY and IBAKY. Upregulated LOC4331761 is “ribulose-phosphate 3-epimerase, chloroplastic-like”. Ribulose-phosphate 3-epimerase is responsible for catalyzing the reversible epimerization of D-ribulose 5-phosphate to D-xylulose 5-phosphate, an intermediate in the pentose phosphate pathway, which generates NADPH and pentoses. LOC4332364 is described as “fructose-1,6-bisphosphatase, chloroplastic-like”. Fructose-1,6-bisphosphatase is an enzyme that catalyzes the reaction from fructose-1,6-bisphosphate into fructose-6-phosphate. Upregulation of LOC4332364 may lead to greater flux of fructose-6-phosphate, the precursor for starch synthesis in chloroplasts.

### Rice co-expressed gene analysis under different backgrounds

The role of the ribosome pathway in plants is mainly involved in protein synthesis and processing. After the mRNA molecule enters the ribosome, the ribosome converts the amino acid sequence into protein through translation. In this study, 17,741 co-expressed genes were screened under three different conditions (CK, CKY, and IBAKY), and 290 co-expressed genes were found to be enriched to the ribosome pathway with the highest significance. Furthermore, 16 highly expressed genes were screened out of the 290 genes in this study, and these genes play an essential role in the ribosome pathway. 

Specifically, this study found that among the 16 co-expressed genes, there were 10 50S ribosomal protein genes, and after using IBAK under salt stress, a total of 9 50S ribosomal protein genes were upregulated compared with CKY. At the same time, this study also found that 4 30S ribosomal protein genes were upregulated under salt stress after using IBAK. These results indicated that the effect of IBAK on gene expression in rice under salt stress deserves attention. These are all essential genes involved in protein synthesis, and their upregulation meant that IBAK may increase protein synthesis in rice under salt stress, vital in maintaining cell function and promoting rice growth and development.

## Conclusions

Spraying IBAK solution under salt stress can adjust the ion balance and improve the antioxidant capacity of rice, which is beneficial to maintain intracellular ion homeostasis and reduce oxidative damage caused by salt stress. In addition, spraying IBAK solution under salt stress can also upregulate the key genes related to photosynthesis, photosynthesis - antenna proteins, plant hormone signal transduction, starch and sucrose metabolism, and pentose phosphate pathway. The upregulation of these genes may help to improve the photosynthetic ability, increase the synthesis of protein, sucrose and starch, and improve the perception and response ability of rice to plant hormones.

## Materials and methods

### Experimental design

This study was conducted at Guangdong Ocean University in 2021. The tested variety was Chaoyou1000. The concentration of IBAK was 80 mg·L^−1^. Both rice and IBAK (high-purity solid particles, 99.99%) in this study were provided by the Physiological and Ecological Research Laboratory of Guangdong Ocean University. Salt concentration was set to 0.28%. The test device was a plastic barrel with an upper diameter: height: lower diameter of 29.3: 38: 26.5 cm, and the bottom was sealed.

After disinfection, the rice seeds were rinsed with tap water. Then, the seeds were sown in the seedling tray and were cultivated outdoors. When the rice seedlings reached the 4-leaf stage, they were transplanted into plastic buckets. The amounts of nitrogen fertilizer (urea), phosphate fertilizer (ammonium dihydrogen phosphate), and potassium fertilizer (potassium chloride) used in each plastic bucket were 2.03 g, 0.9 g, and 1.12 g, respectively. The nitrogen fertilizer was applied according to the ratio of base fertilizer: tillering fertilizer: panicle fertilizer = 4:3:3. All of the phosphate fertilizer was used as base fertilizer, while the potassium fertilizer was applied according to the ratio of base fertilizer: panicle fertilizer = 7:3.

IBAK solution was sprayed on the rice leaves at the early jointing stage. One week after spraying, the latest fully expanded leaf on the main stem was selected for subsequent measurement and analysis of the relevant indicators.

### Determination of Na^+^, K^+^, and Cl^−^ in rice leaves

The rice leaves were dried at 105℃ for 0.5 h and then were at 70℃ to constant weight. The dried samples were pulverized, sieved, and stored for later use.

0.15 g of the finely crushed sample was mixed with 6 mL of nitric-perchloric acid (mixed at a ratio of 4:1) and digested in a digestion furnace until it became clear. The solution was then diluted to 100 mL in a volumetric flask and filtered to measure K^+^ and Na^+^ content. The Na^+^ content in rice leaves was determined using an Inductively Coupled Plasma Emission Spectrometer. The K^+^ content was determined by digestion with nitric-perchloric acid and measured using a flame photometer.

A 0.5 g sample was extracted with boiling water for 1.5 h and then made up to 100 mL after filtration for measurement. The Cl^−^ content was determined by the silver nitrate titration method.

The determination of Na^+^, K^+^, and Cl^−^ content in rice leaves was completed by the South Subtropical Crop Research Institute, China Academy of Tropical Agricultural Sciences.

### Determination of Pn, Tr, intercellular carbon dioxide concentration (Ci), and Gs 

The portable photosynthetic instrument Li6800 (LI-COR, USA) was used to measure the rice photosynthetic parameters Pn (µmol·m^−2^·s^−1^), Tr (mmol·m^−2^·s^−1^), Ci (µmol·mol^−1^), and Gs (mol·m^−2^·s^−1^) one week after the application of IBAK.

### Determination of SOD, POD, CAT, and APX activity

0.5 g of leaves were ground into homogenate with pre-cooled phosphate buffer solution (pH 7.8, 50 Mm). The mixture was centrifuged at 10,000 g for 10 min at 4 ◦C, and the supernatant was used to determine enzyme activity. The determination of SOD, POD, CAT, and APX activity was based on the method described by Huang et al. [11].

### Determination of GSH and soluble protein contents

The determination of GSH content was based on the method described by Yan et al. [33]. The determination of soluble protein content was based on the method described by Kučerová et al. [34].

### Transcriptome sequencing

Transcriptome sequencing was carried out by Novogene Co., Ltd. Total RNA was used as input material for the RNA sample preparations. After the library construction was completed, the Qubit2.0 Fluorometer was used for preliminary quantification. The library was diluted to 1.5 ng·ul^−1^, and then the Agilent 2100 bioanalyzer was used to detect the insert size of the library. After the insert size met expectations, qRT-PCR accurately quantified the effective concentration of the library to ensure the library quality. GO terms with Padj less than 0.05 were considered significantly enriched by differentially expressed genes. For KEGG pathway enrichment, Padj less than 0.05 was considered as the threshold of significant enrichment. KEGG pathway map images has been licensed for use in this study [35–37].

### Quantitative real-time PCR (qPCR)

qRT-PCR was performed and completed by Sangon Biotech (Shanghai) Co., Ltd. qRT-PCR is to add a fluorescent dye (SYBR green I) to the PCR reaction system, use the accumulation of fluorescence signals to monitor the PCR process in real time, and conduct quantitative analysis of the initial template. The steps performed included primer design, RNA extraction, reverse transcription, and fluorescence quantitative PCR detection. Primer Premier 5.0 software was used in this study to design primers. The RNA extraction procedure was based on the UNIQ-10 column Trizol total RNA extraction kit (B511321).

Relative quantification was based on the mathematical operation of 2^−(ΔΔCt)^ between the CT value of the internal reference gene and the CT value of the gene to be tested to obtain the fold difference in expression levels between samples. The internal reference gene is *UBQ10* (Table S11).

### Statistical analysis

The data was subjected to ANOVA using SPSS 22. The figures were created using Origin 2021.

### Supplementary Information

**Additional file 1:** **Fig. S1.** K^+^, Na^+^, and Cl^-^ content in rice leaves. CK, under freshwater conditions; CKY, under salt stress conditions; IBAKY, IBAK treatment under salt stress conditions. **Fig. S2.** Photosynthesis-related indicators in rice leaves. CK, under freshwater conditions; CKY, under salt stress conditions; IBAKY, IBAK treatment under salt stress conditions. Pn, net photosynthetic rate; Tr, transpiration rate; Ci, intercellular carbon dioxide concentration; Gs, stomatal conductance. **Fig. S3.** Antioxidant enzyme activities in rice leaves. CK, under freshwater conditions; CKY, under salt stress conditions; IBAKY, IBAK treatment under salt stress conditions. **Fig. S4.** GSH and soluble protein contents in rice leaves. CK, under freshwater conditions; CKY, under salt stress conditions; IBAKY, IBAK treatment under salt stress conditions. **Table S1.** Sample sequencing data quality summary. **Table S2.** Reference genome alignment statistics. **Table S3.** Validation of selected genes using qRT-PCR. **Table S4.** Statistical results of DEGs. **Table S11.** Internal control gene.

**Additional file 2:** **Table S5.** DEGs statistics in the CKY vs. CK comparison group.

**Additional file 3:** **Table S6.** DEGs statistics in the IBAKY vs. CKY comparison group.

**Additional file 4:** **Table S7.** GO enrichment analysis in the CKY vs. CK comparison group.

**Additional file 5:** **Table S8.** GO enrichment analysis in the IBAKY vs. CKY comparison group

**Additional file 6:** **Table S9.** KEGG enrichment analysis in the CKY vs. CK comparison group.

**Additional file 7:** **Table S10.** KEGG enrichment analysis in the IBAKY vs. CKY comparison group.

## Data Availability

The datasets generated and/or analysed during the current study are available in the GSA repository (https://bigd.big.ac.cn/gsa/browse/CRA012129).
