# Supplementary material for: Transcriptomics and physiology reveal the mechanism of potassium indole-3-butyrate (IBAK) mediating rice resistance to salt stress
Source: BMC Plant Biol. 2023 Nov 16;23:569. doi: 10.1186/s12870-023-04531-1 (PMC10652493; doi:10.1186/s12870-023-04531-1)
Supplement: Supplementary file 1 — Additional file 1: Fig. S1. K+, Na+, and Cl- content in rice leaves. CK, under freshwater conditions; CKY, under salt stress conditions; IBAKY, IBAK treatment under salt stress conditions. Fig. S2. Photosynthesis-related indicators in rice leaves. CK, under freshwater conditions; CKY, under salt stress conditions; IBAKY, IBAK treatment under salt stress conditions. Pn, net photosynthetic rate; Tr, transpiration rate; Ci, intercellular carbon dioxide concentration; Gs, stomatal conductance. Fig. S3. Antioxidant enzyme activities in rice leaves. CK, under freshwater conditions; CKY, under salt stress conditions; IBAKY, IBAK treatment under salt stress conditions. Fig. S4. GSH and soluble protein contents in rice leaves. CK, under freshwater conditions; CKY, under salt stress conditions; IBAKY, IBAK treatment under salt stress conditions. Table S1. Sample sequencing data quality summary. Table S2. Reference genome alignment statistics. Table S3. Validation of selected genes using qRT-PCR. Table S4. Statistical results of DEGs. Table S11. Internal control gene. [file 12870_2023_4531_MOESM1_ESM.docx]

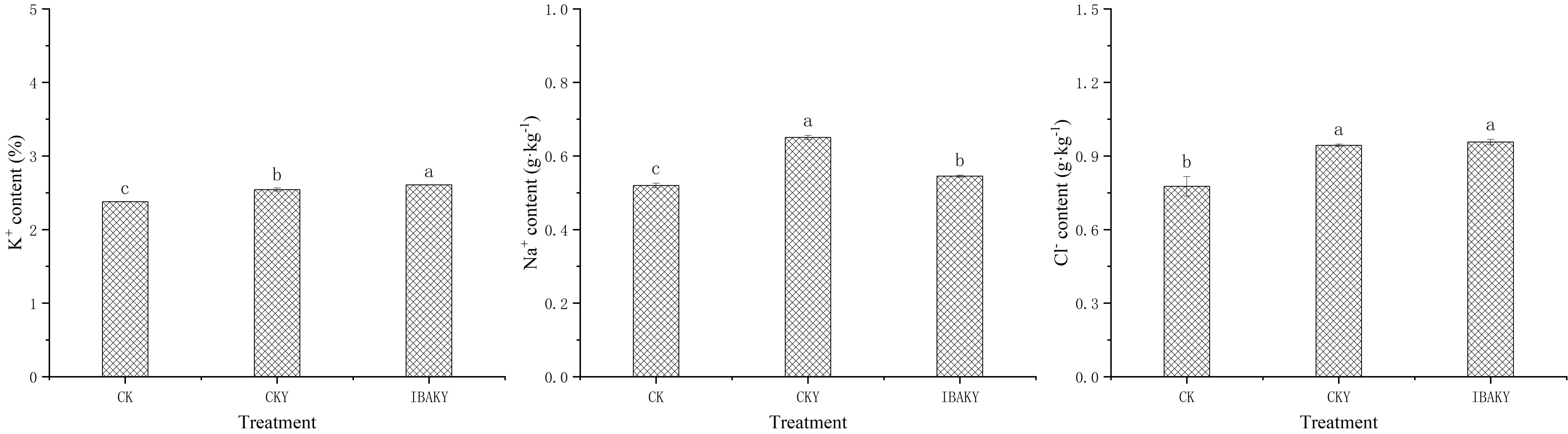


Fig.S1 K^+^, Na^+^, and Cl^-^ content in rice leaves. CK, under freshwater conditions; CKY, under salt stress conditions; IBAKY, IBAK treatment under salt stress conditions.


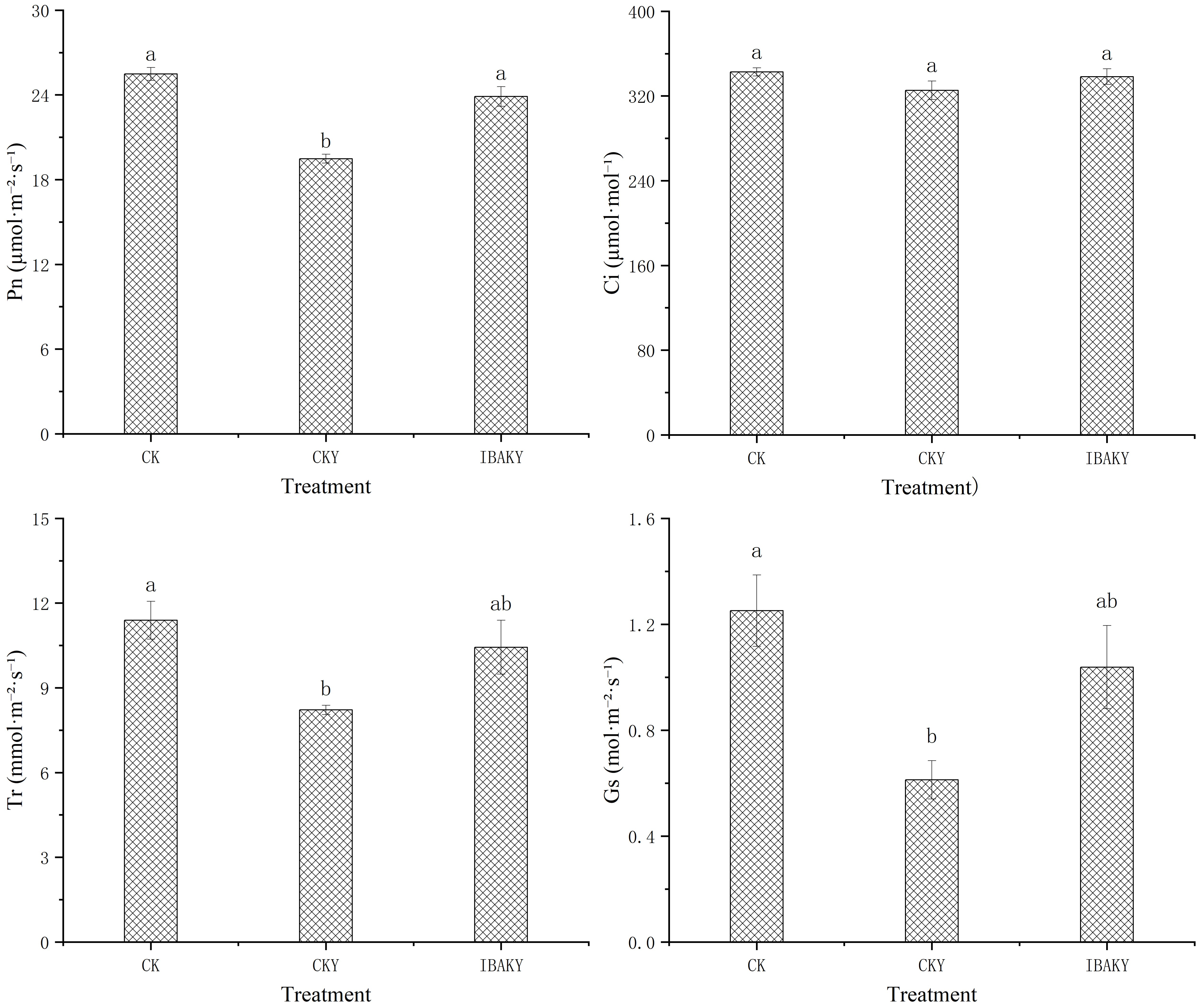


Fig.S2 Photosynthesis-related indicators in rice leaves. CK, under freshwater conditions; CKY, under salt stress conditions; IBAKY, IBAK treatment under salt stress conditions. Pn, net photosynthetic rate; Tr, transpiration rate; Ci, intercellular carbon dioxide concentration; Gs, stomatal conductance.


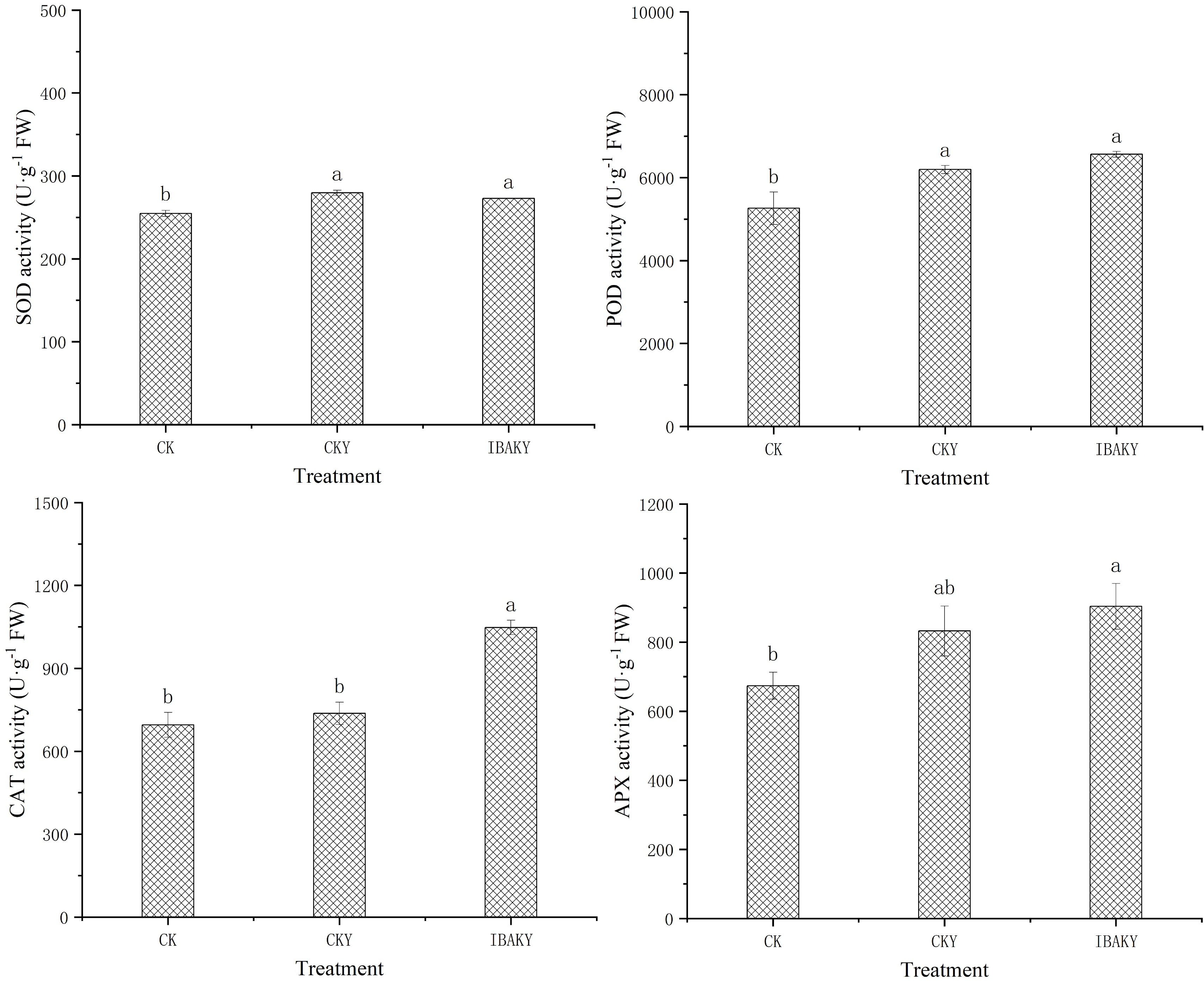


Fig.S3 Antioxidant enzyme activities in rice leaves. CK, under freshwater conditions; CKY, under salt stress conditions; IBAKY, IBAK treatment under salt stress conditions.


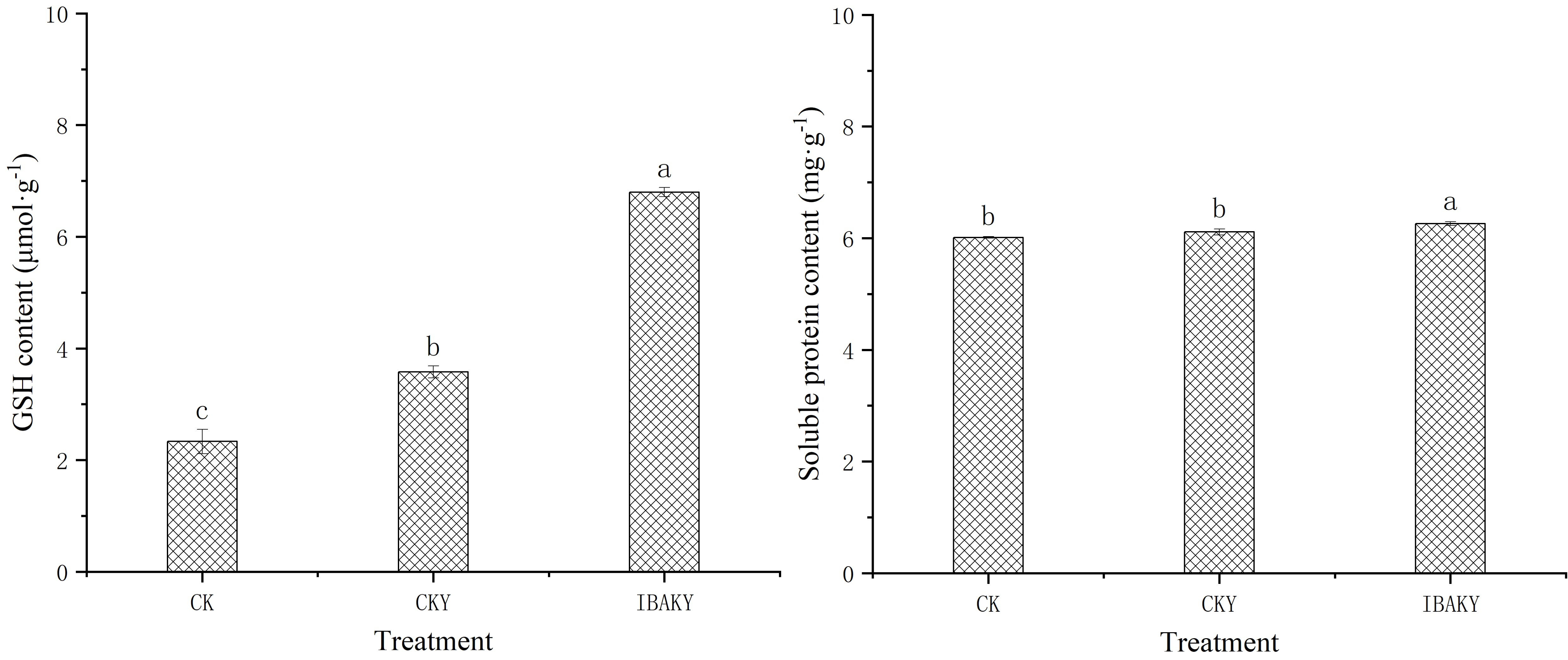


Fig.S4 GSH and soluble protein contents in rice leaves. CK, under freshwater conditions; CKY, under salt stress conditions; IBAKY, IBAK treatment under salt stress conditions.

**Table.S1 Sample sequencing data quality summary**

| Sample | Raw reads | Raw bases | Clean reads | Clean bases | Error rate | Q20 | Q30 | GC pct |
| --- | --- | --- | --- | --- | --- | --- | --- | --- |
| CK1 | 46292564 | 6.94G | 44902752 | 6.74G | 0.03 | 97.55 | 93.47 | 54.12 |
| CK2 | 47073982 | 7.06G | 45658472 | 6.85G | 0.03 | 97.72 | 93.82 | 54.31 |
| CK3 | 43983652 | 6.6G | 42332054 | 6.35G | 0.03 | 97.35 | 92.98 | 53.96 |
| CKY1 | 41712198 | 6.26G | 41008272 | 6.15G | 0.03 | 97.51 | 93.37 | 53.79 |
| CKY2 | 44627760 | 6.69G | 43878438 | 6.58G | 0.03 | 97.54 | 93.41 | 53.44 |
| CKY3 | 46481184 | 6.97G | 45561666 | 6.83G | 0.03 | 97.21 | 92.72 | 53.25 |
| IBAKY1 | 41695236 | 6.25G | 40817494 | 6.12G | 0.03 | 97.37 | 93.03 | 54.44 |
| IBAKY2 | 44474688 | 6.67G | 43521206 | 6.53G | 0.03 | 97.47 | 93.24 | 54.19 |
| IBAKY3 | 44489446 | 6.67G | 43160102 | 6.47G | 0.03 | 97.29 | 92.84 | 54.13 |

CK, under freshwater conditions; CKY, under salt stress conditions; IBAKY, IBAK treatment under salt stress conditions.

**Table.S2 Reference genome alignment statistics**

| Sample | Total reads | Total map | Unique map |
| --- | --- | --- | --- |
| CK1 | 44902752 | 40906197(91.1%) | 39919374(88.9%) |
| CK2 | 45658472 | 41714353(91.36%) | 40700621(89.14%) |
| CK3 | 42332054 | 38359983(90.62%) | 37501760(88.59%) |
| CKY1 | 41008272 | 37410668(91.23%) | 36533555(89.09%) |
| CKY2 | 43878438 | 40111963(91.42%) | 39139586(89.2%) |
| CKY3 | 45561666 | 41606924(91.32%) | 40503441(88.9%) |
| IBAKY1 | 40817494 | 37207102(91.15%) | 36153645(88.57%) |
| IBAKY2 | 43521206 | 39729601(91.29%) | 38704762(88.93%) |
| IBAKY3 | 43160102 | 39317033(91.1%) | 38338411(88.83%) |

CK, under freshwater conditions; CKY, under salt stress conditions; IBAKY, IBAK treatment under salt stress conditions.

**Table.S3 Validation of selected genes using qRT-PCR.**

| ID | Primer sequence | Treatment | Relative expression level | FPKM |
| --- | --- | --- | --- | --- |
| 4330957 | F 5' GACATCAAGCCATCCAACCT 3'  R 5' GTCCCGACGGAGGAGTTG 3' | CK | 1 | 38.52057192 |
|  |  | CKY | 0.886872 | 18.16136587 |
|  |  | IBAKY | 1.794375 | 56.26790555 |
| 4325998 | F 5' TACTTCCATCCTGTTCTTCTTCCT 3'  R 5' GGCAGCCTCACGATGTTGT 3' | CK | 1 | 252.1110206 |
|  |  | CKY | 0.545615 | 114.8074513 |
|  |  | IBAKY | 20.63612 | 344.8863247 |
| 4345657 | F 5' TGCTGGGATCACAATTCTACC 3'  R 5' TTCGCCTCCACCACCTCT 3' | CK | 1 | 54.89206607 |
|  |  | CKY | 0.822869 | 28.80532615 |
|  |  | IBAKY | 1.947213 | 71.82303593 |

CK, under freshwater conditions; CKY, under salt stress conditions; IBAKY, IBAK treatment under salt stress conditions.

**Table.S4 Statistical results of DEGs**

| Exported data | | | | |
| --- | --- | --- | --- | --- |
| **Compare** | **All** | **Up** | **Down** | **Threshold** |
| CKY vs CK | 1126 | 401 | 725 | DESeq2 pvalue<=0.05 \|log2FoldChange\|>=0.0 |
| IBAKY vs CKY | 2276 | 1017 | 1259 | DESeq2 pvalue<=0.05 \|log2FoldChange\|>=0.0 |

CK, under freshwater conditions; CKY, under salt stress conditions; IBAKY, IBAK treatment under salt stress conditions.

**Table.S11 Internal control gene**

| Gene | Primer sequence |
| --- | --- |
| *UBQ10* | F 5' TTCGTGAAGACCTTGACTGGG 3' |
|  | R 5' CACCAAACTGGCTGATTACTGAC 3' |
